# Supplementary figures and images for: Influence of Geographical Orchard Location on the Microbiome from the Progeny of a Pecan Controlled Cross
Source: Plants (Basel). 2023 Jan 12;12(2):360. doi: 10.3390/plants12020360 (PMC9862047; doi:10.3390/plants12020360)

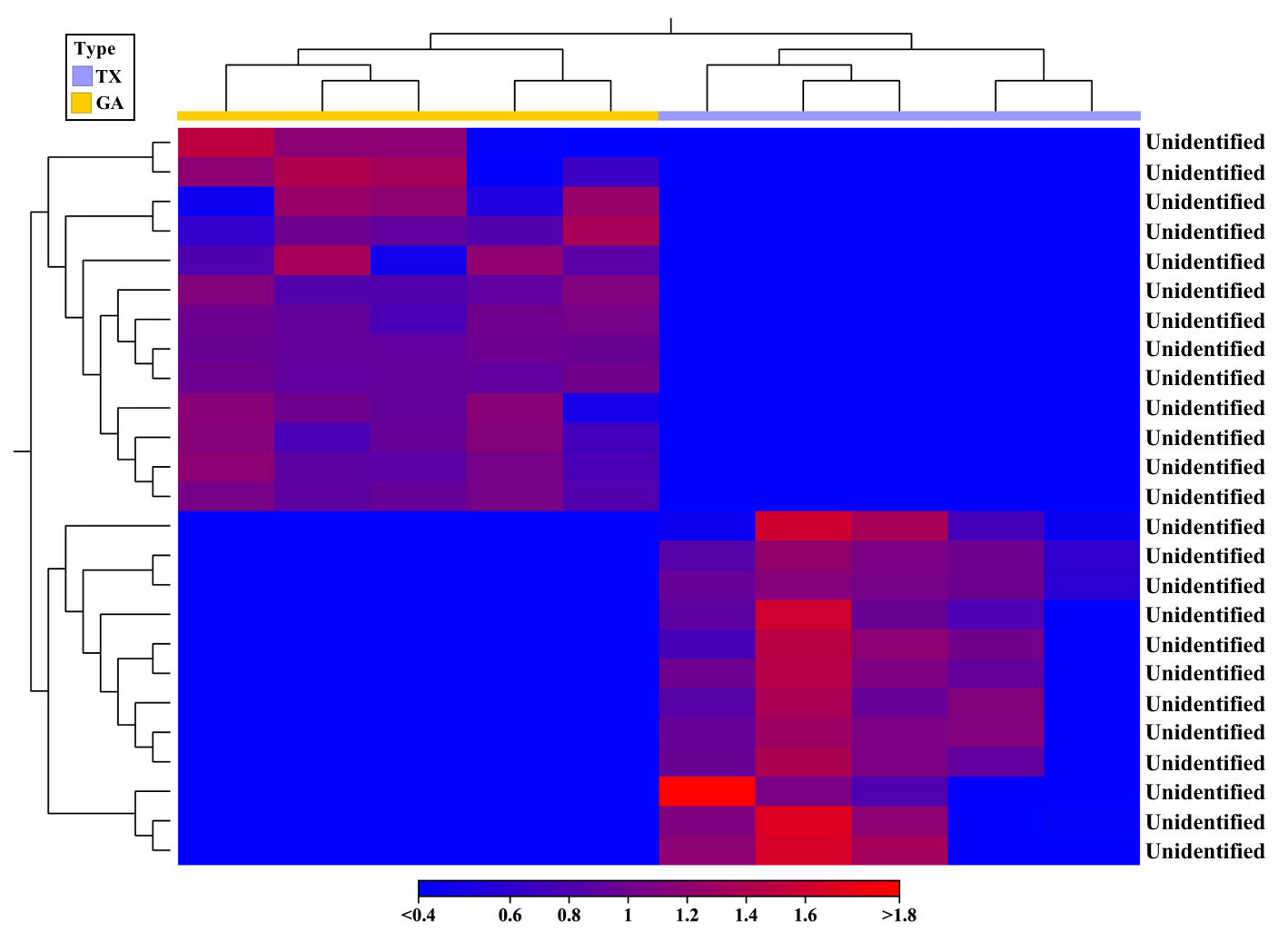

Supplement: Supplementary file 1 [file plants-12-00360-s001.zip › Figure S1.jpeg]

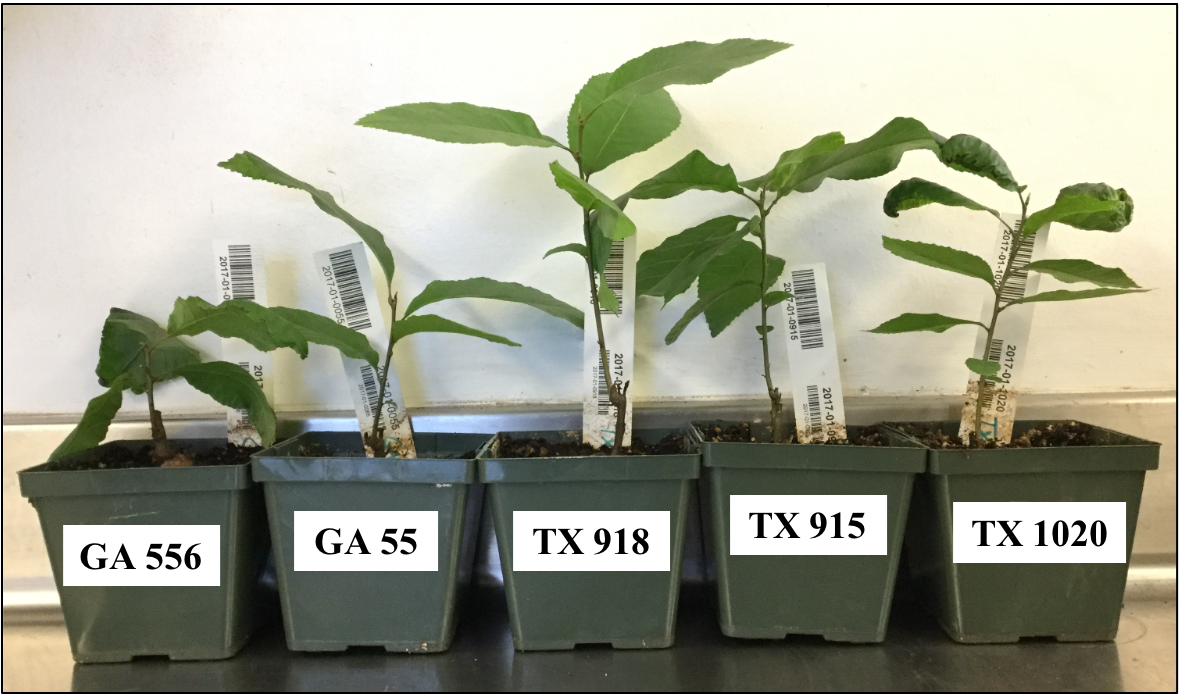

Supplement: Supplementary file 1 [file plants-12-00360-s001.zip › Figure S2.jpeg]
